# Living probiotics-loaded wound matrices prepared by microchip electrospinning

**DOI:** 10.1016/j.mtbio.2025.102403

**Published:** 2025-10-10

**Authors:** Oksana Gerulis, Georg-Marten Lanno, Marta Putrinš, Marilin Moor, Beata Niemczyk-Soczynska, Tomasz Kowalczyk, Slawomir Blonski, Tanel Tenson, Piotr M. Korczyk, Karin Kogermann

**Affiliations:** a Institute of Pharmacy, University of Tartu, Nooruse 1, 50411, Tartu, Estonia; b Institute of Computer Science, University of Tartu, Narva mnt 18, Tartu, 51009, Tartumaa, Estonia; c Institute of Fundamental Technological Research, Polish Academy of Sciences, Pawinskiego 5B, 02-106, Warsawa, Poland; d Institute of Technology, University of Tartu, Nooruse 1, 50411, Tartu, Estonia

**Keywords:** Microchip electrospinning, Microfluidics, Electrospinning, Probiotics, Wound infection

## Abstract

Live biotherapeutic products are an emerging novel class of products currently under development to be used for the treatment of clinical challenges such as atopic dermatitis, acne, chronic wounds. Several methods of encapsulation are available to preserve the viability of probiotic bacteria in various harsh environmental conditions. In this work, an innovative microchip electrospinning is developed, which combines microfluidics microchip with electrospinning and enables the preparation of fiber matrices comprising living and functional encapsulated bacteria capable of producing antimicrobial substances. The bacteria are encapsulated into microcapsules, which are immediately within the same process electrospun into hydrophobic fibers. Using confocal microscopy and staining samples with fluorescent dyes, it is confirmed that probiotics are present in fibers. The average concentration of probiotics is 10^6^ bacteria/cm^2^ in a 1 mm thick matrix. Using an agar overlay assay, it is determined that incorporated probiotics retain their functionality and antimicrobial activity against wound pathogens. This evidence confirms that the electrospun fibers containing microcapsules allow two-way diffusion of substances through pores in fibers (e.g., nutrients in, produced substances out) and support the viability of entrapped bacteria. The electrospun probiotics-loaded fiber matrix developed has potential to be used as a drug delivery system for wound infection treatment.

## Highlights

• Microchip electrospinning technology is setup and allows producing living probiotics loaded fibrous wound matrices.
• The electrospun fibers containing microcapsules allow two-way diffusion of substances through pores in fibers. ∗Microchip electrospun microcapsule loaded fibrous wound matrices support the viability of entrapped probiotic bacteria.
• Probiotics-loaded wound matrices revealed good mechanical properties, wettability, sufficient short-term storage stability. ∗Desired antimicrobial properties against relevant wound pathogens can be obtained.
• Our findings may help in developing novel treatments for wound infections.

## Graphical abstract

**Figure:**
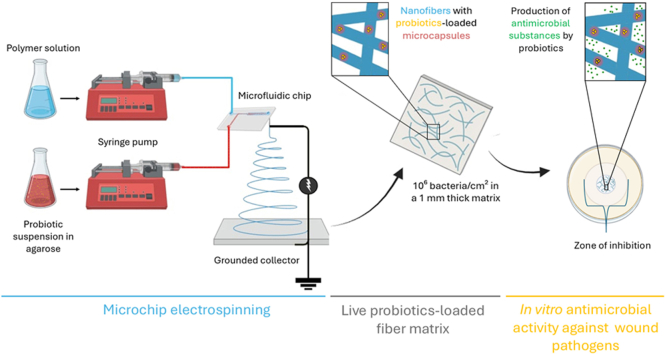

## 1 Introduction

Biomaterials are materials designed to interact with the body to replace, support, or restore impaired functions, improving the health and quality of life while being compatible with biological systems [1]. They can be of natural or synthetic origin. Biomaterials are specifically designed to perform a defined function within biological systems and their biocompatibility depends on the defined application, since biocompatibility for one application may not be biocompatible in another [2].

During recent years, various novel biomaterials have been developed. When active pharmaceutical ingredients (APIs) are incorporated into the material, novel and more effective drug delivery systems are developed. The materials containing living cells (responsive function) and polymeric matrices (scaffolding function) and, thus, can be designed as active and responsive biomaterials [3,4]. Biomaterials can contain mammalian cells, bacteria, microalgae, yeasts, viruses and plant cells [5].

Probiotics are live microorganisms that, when administered in sufficient quantities, are beneficial to health, e.g., provide balance in gut microbiome [6]. Probiotics are governed by stringent regulatory definitions, especially when used for therapeutic purposes [7,8]. Not every bacterium can be considered as a probiotic. Most probiotic bacteria belong to the highly diverse families *Lactobacillus* spp. and *Bifidobacterium* spp., which are important in human nutrition and biotechnology due to their ability to produce organic acids and other biologically active substances, such as antimicrobial peptides (AMPs) or hydrogen peroxide [9,10]. In recent years, live biotherapeutic products (LBPs) have been introduced and are continuously evolving into various health-related applications [11]. Unlike traditional probiotics, which are naturally isolated from the environment and proven safe for food and therapeutic use, LBPs often involve genetically modified bacteria to enhance their functionality [12].

Probiotics are currently under the development into several LBPs to be used for the topical treatment of clinical challenges such as atopic dermatitis [13], acne [14], or chronic wounds [15]. In the coming years, novel LBPs are expected to be utilized more widely. Their advantage lies in the ability to engineer bacteria to produce the necessary substances for treatment, rather than relying on probiotics that naturally produce the molecules. Various physicochemical and biological factors may largely affect the stability and *in vivo* performance of probiotics. Probiotics need to preserve their viability under environmental stress (oxidation, reduction, humidity, temperature, pH changes), and stability in biological fluids [16]. Thus, in order to preserve their viability and functionality in demanding environments, the use of probiotics for the local treatment of wound infections faces various challenges.

The development of innovative probiotic containing systems has shown potential to increase their efficacy in topical medical applications. Thus, there is a great interest in the design and fabrication of systems that can encapsulate, protect, deliver and release probiotics [17]. One of the shielding technologies is the encapsulation of bacteria which is needed to protect the bacteria in the wound environment (e.g. phagocytosis) and prolong the action time of the wound dressing. Encapsulation is a technique where cells are entrapped in a semipermeable polymeric membrane. Microencapsulation methods are the most widely used methods [18] for the protection and preparation of delivery vehicles for living bacterial cells (including probiotics) [19]. Several methods such as spray drying, extrusion, emulsion techniques, electrospinning/electrospraying are effective in encapsulating living microbes into polymeric materials [20,21]. The advantages of the electrospinning/electrospraying techniques are the production of very thin fibers or capsules to the order of few nanometers with large surface areas [22,23]. Biologicals, like cells, proteins, and small molecules like antibiotics, and other drugs have been encapsulated via electrospinning. Coaxial and emulsion electrospinning/electrospraying techniques produce core-shell structures, which have shown promising results in protecting living probiotics during encapsulation [24,25]. The high surface area of the cargo-loaded nanofiber matrices, ease of separation, protection from harsh external conditions, and reduced susceptibility to contamination by foreign organisms enable excellent delivery characteristics [20,26]. In conjunction with their excellent flexibility and porosity, nanofiber matrices with encapsulated biologics hold great potential in wound dressings, protective coatings, and biological scaffolds [26]. Recently, also microfluidic methods have been adopted for encapsulating and protecting bacteria from damaging agents [27]. Whilst, multi-fluid electrospinning and the related multi-chamber nanostructures provide numerous advantages in the development of novel biomaterials [27,28].

Although both microfluidics and electrospinning have been used for the encapsulation of living probiotics, not much data are available showing their use in combination and applied for preparing probiotics-loaded fiber matrices. One example of microchip electrospinning hybrid method's potential for producing multicompartmental fibrous films has been recently reported, but not specifically for the encapsulation of probiotics [29]. Microchip electrospinning method should not be confused with microfluidic spinning method, which is known to exist for more than 10 years [30,31]. In the latter case, the preparation of probiotic delivery systems is mainly by injection or coacervation methods. Unfortunately, all these methods have some disadvantages and challenges, like toxic impact of solvent on the probiotics, loss of functionality, low viability of probiotics and short shelf life of the final product [32,33]. Interestingly, most of the existing literature reports the electrospinning of water-soluble fiber matrices loaded with living probiotics [21] and do not allow for prolonged activity in the wound. Selection of the formulation components is of importance in order to obtain a suitable delivery system for probiotics. Both natural polymers [34] and synthetic polymers [35] can be used for preparing wound dressing materials. Biodegradable polymers (e.g. polycaprolactone, polylactide etc) which exhibit poor aqueous solubility are of interest to keep the probiotics protected within the dressing and allow simultaneous contamination protection for the wound protection for the wound.

Novel strategies and delivery platforms are needed that protect the probiotics from destruction enabling effective delivery to the wound. These approaches enable us to produce living probiotics-loaded systems for the local treatment of wound infections. The aim of the present study is to develop novel fiber matrices containing living probiotic bacteria, preserving their viability and functionality, by using microchip electrospinning technology. The main focus is on the formulation of living probiotics-loaded water insoluble wound matrices, microchip electrospinning allowing *in situ* formation of microcapsules loaded with probiotics and their simultaneous electrospinning into fibers and the characterization of the matrices in biorelevant assays to prove their antibacterial efficacy.

## 2 Results

### 2.1 Formulation development for the microchip electrospinning

**Fig. 1:**
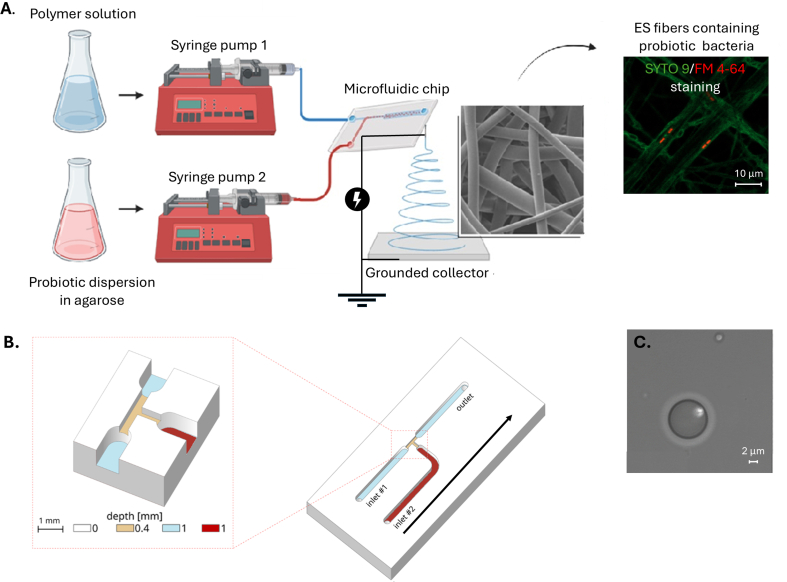
**A.** Schematic illustration of microchip electrospinning set-up together with the scanning electron microscopy (SEM) micrograph of obtained electrospun (ES) living probiotics-loaded fiber matrix and confocal fluorescence microscopy (CFM) micrograph where the bacteria are shown in red within fibers and fibers in green, samples stained using FM 4–64 and SYTO 9, respectively; **B.** Polydimethylsiloxane (PDMS) chip design, the microfluidic chip (PDMS) included two inlet channels (inlet #1 and inlet #2) — inlet #1 connected to a syringe with the PLC/PEO polymer solution and inlet #2 to a syringe containing the agarose-bacterial dispersion. These channels converged into a common outlet channel, which was connected to a metal needle (21G). The electrospinning voltage was applied to the needle tip, and fibers were collected on a grounded collector plate at a distance of 13 cm. Flow direction is pointed out with an arrow. **C**- microcapsule with labelled *E. coli* BW25113 micrograph. (For interpretation of the references to color in this figure legend, the reader is referred to the Web version of this article.)

Preliminary testing included the formulation of microcapsules and loading of microcapsules into the electrospun fibers (Supporting Information). Since the full preparation process consisted of several steps which need separate validation (e.g. purification, filtration), then microchip electrospinning was developed and tested in the present study (Fig. 1).

The method allowed the *in situ* preparation of microcapsules suspended in a polymer solution, which was then directly electrospun into fibers (Fig. 1). A chip made of polydimethylsiloxane (PDMS) was used and different chip designs (various channel sizes, i.e. width, height and length) were tested from which the final design was selected (Fig. 1B). More details provided in the *Experimental* section. The selection was made when reproducible microcapsule formation and optimal electrospinning (e.g. microcapsules with homogeneous size, smooth and reproducible electrospinning process, e.g. no major dripping of electrospinning polymer solution, consistent stream) was obtained. Alternative formulations (sodium alginate together with CaCl_2_ solution *vs* agarose) and concentrations of agarose aqueous solutions (0.625–2.5 w/w%) were all tested in preliminary experiments (Supporting Information). The 0.625 w/w% agarose in aqueous solution showed the best results and was the selected formulation for microchip electrospinning to produce microcapsules and microcapsule-loaded fiber systems. Microcapsule formation and incorporation of labelled bacteria was observed visually in a microfluidic chip and morphology and size of microcapsules determined using optical microscopy (Fig. 1C). Syringe pumps were employed to maintain a constant flow of the two immiscible phases, thereby ensuring stable and reproducible conditions for both (i) encapsulation of bacteria within the polymer solution, and (ii) continuous outflow of the resulting suspension from the chip into the nanofiber formation region.

**Fig. 2:**
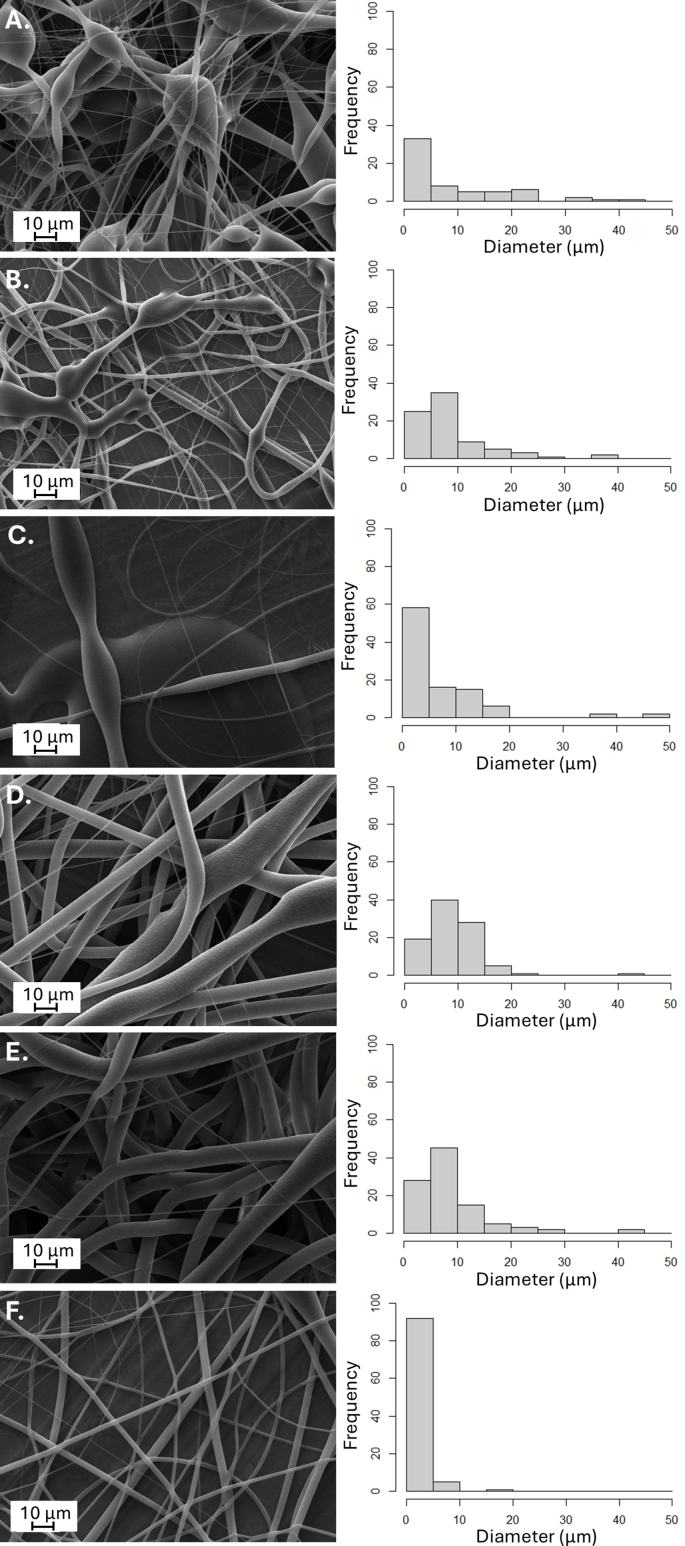
Effect of PLC concentration and voltage on electrospun PLC fibers morphology and diameter. Scanning electron microscopy (SEM) micrographs of 12.5 w/w% PLC fibers electrospun from Me_2_CO_3_ with 0.8 mL/h flow rate and voltages of **A** – 13 kV; **B** – 15 kV; **C** – 17 kV, histograms showing the fiber diameter distributions. SEM micrographs of 15 w/w% PLC fibers electrospun from Me_2_CO_3_ with 0.4 mL/h flow rate and voltages of **D** – 11 kV; **E** – 13 kV; **F** – 15 kV, histograms showing the fiber diameter distributions. Electrospinning conducted using microchip electrospinning set-up.

The solvent and polymer selection for electrospinning was conducted keeping in mind the presence of living probiotics and final wound dressing application, and the rheological behavior of the formulations tested to understand the effect of viscosity to the microchip electrospinning. The selection of solvent was challenging as not all organic solvents are compatible with the chip material used (PDMS). Other solvents were also tested (Table S1, Supporting Information), but Me_2_CO_3_ (dimethyl carbonate) gave the best results considering the chip and polymer materials, electrospinnability and viability of bacteria. Initially pristine polycaprolactone (PCL), molecular weights of 80 000 vs 120 000 g/mol, was tested (15 w/w% concentration), but PCL did not dissolve in the selected solvent Me_2_CO_3_, hence PCL was excluded from further study. Poly(L-lactide-co-glycolide (PLC), another hydrophobic polymer dissolved in Me_2_CO_3_ and was tested in different concentrations (5 w/w% to 20 w/w%) for microchip electrospinning. Fibers with lower polymer concentration, for example 12.5 w/w% PLC, had beads on the fiber and fibers exhibited very inhomogeneous diameter distribution (Fig. 2A, B and 2C).

The mean diameter of 12.5 w/w% PLC fibers using 15 kV was 5.38 ± 4.78 μm. Higher PLC concentrations (15 w/w%) resulted in fiber mean diameter of 4.61 ± 2.85 μm. The uniform size is important outcome of successful electrospinning. The voltage effect was studied (tested range from 11 kV up to 15 kV) and the suitable voltage for 15 w/w% PLC in Me_2_CO_3_ solution was 13 kV (Fig. 2). It was observed that higher voltages resulted in smaller and more homogeneous fiber diameters, but increasing the voltage further made the electrospinning process discontinuous, and electrospraying occurred.

**Fig. 3:**
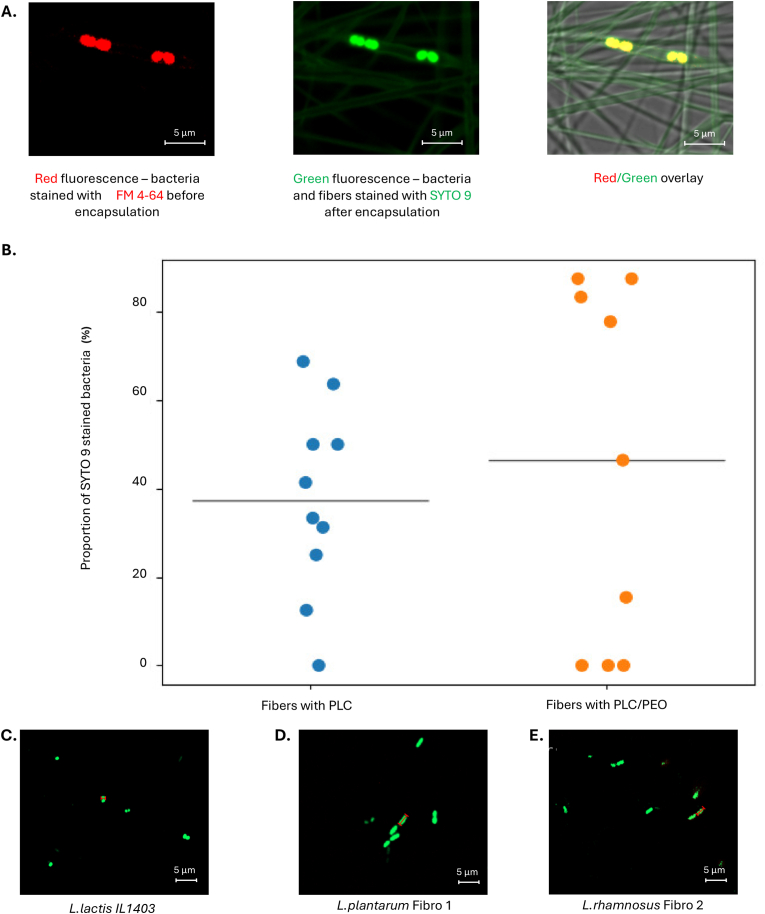
**A.** Representative zoom-in of confocal fluorescence microscopy (CFM) micrographs of electrospun PLC/PEO fiber matrices (with a size of original image 67.48 × 67.48 μm) consisting of 0.625 w/w% agarose microcapsules with FM 4-64-stained (red fluorescence) living *L. lactis* IL1403 on M17 + lactose+ 0.5 w/v% glucose agarose plates without SYTO 9 and with SYTO 9 (green fluorescence) and overlay images (red and green fluorescence). The plates were kept at 30 °C after electrospinning for specified time periods (24 h and 48 h). **B.** Proportion of SYTO 9-stained *L. lactis* IL1403 bacteria in two different formulations: PLC fiber matrices and PLC/PEO fiber matrices. Each dot represents one analyzed microscopy sample (on average, 17 bacteria per sample). Formulations were statistically compared (Welch *t*-test, p-value = 0.669, not statistically significant). **C.** CFM micrographs showing the morphology of lactic acid bacteria - *L. lactis* IL1403, **D.***L. plantarum* Fibro 1 and **E.***L. rhamnosus* Fibro 2, samples stained with SYTO 9 (green fluorescence). Key: scale bar: 5 μm. The red line indicates the size of the lactic acid bacteria. (For interpretation of the references to color in this figure legend, the reader is referred to the Web version of this article.)

PLC in Me_2_CO_3_ formulation (concentrations ranging from 13 w/w% PLC to 15 w/w% PLC) with agarose was suitable for microchip electrospinning allowing to produce agarose microcapsule-loaded fibers. Different process parameters such as flow rate, voltage and distance were also tested for microchip electrospinning, and optimal conditions developed. Although, it was also possible to obtain probiotics-loaded fiber matrices with pristine PLC and agarose formulation, these fibers did not allow the desired transport rates of substances into and out from the fibers (Fig. 3 and Fig. S2, Supporting Information). Therefore, polyethylene oxide (PEO) was included into the formulation to increase the nanoporosity of the fibers in aqeous conditions and increase the exchange of substances in and out from the fibers. The diffusion of green-fluorescent nucleic acid stain (SYTO 9) into the fibers containing microcapsules and its capability to enter into bacterial cells and emit green fluorescence upon binding to DNA was tested (Fig. 3A and B). Bacteria were initially (before electrospinning) stained using membrane stain FM 4–64 which stained them red. The visualization was performed using confocal fluorescence microscopy (CFM). Both bacteria (inside the fibers and outside the fibers) were stained with SYTO 9. The results show that all bacteria stained with FM 4–64 were red, but if SYTO 9 was added, the bacteria also emitted green fluorescence (Fig. 3A). The SYTO 9 stain was able to diffuse through the pores in the fibers and microcapsules. Although a trend toward higher % of bacteria stained with SYTO 9 and giving green fluorescent signal in addition to red in PLC/PEO samples was observed (Fig. 3B), the difference compared to pristine PLC did not reach statistical significance (p = 0.069). The lack of significance may also reflect limitations in the sensitivity of fluorescence-based bacterial quantification, as bacterial localization (non-homogeneous distribution) may not directly correspond to pore size distribution. Also, this may be due to the limited sample size, which reduces statistical power. Despite this, it was concluded that the PLC/PEO fibers have sufficient nanoporosity which is also relevant for the future wound infection treatment application, as we assume that antimicrobial peptides and metabolites will also diffuse. The selected formulation for microchip electrospinning to produce microcapsules and living probiotics-loaded microcapsule fiber systems was as follows. One solution consisted of 12 w/w% PLC and 0.3 w/w% PEO dissolved in Me_2_CO_3_, while the other was an aqueous solution containing 0.625 w/w% agarose. The mean viscosity of the two polymer solutions was 721 ± 147 cP and this allowed suitable properties for microchip electrospinning. This formulation was further fully characterized and tested with relevant probiotic strains. Pristine 12 w/w% PLC/0.3 w/w% PEO fiber matrix with no agarose microcapsules and the same fiber matrix with agarose microcapsules, but not loaded with probiotics, were used as controls.

### 2.2 Microchip electrospinning to prepare living lactic acid bacteria-loaded fibers

For the preparation of functional electrospun fiber matrices as living biomaterials, different lactic acid bacteria (LAB) *- L. lactis* IL1403, *L. plantarum* Fibro 1 and *L. rhamnosus* Fibro 2 were used. These are Gram positive, lactic acid-producing bacteria and classified by the FDA as GRAS bacteria [36]. *L. plantarum* Fibro 1 and *L. rhamnosus* Fibro 2 are recommended strains in according to the European Food Safety Authority (EFSA) and qualified presumption of safety (QPS) [37]. *L. lactis* IL1403 has spherical shape (Fig. 3C) with diameter 0.91 ± 0.16 μm. This strain is preferentially grown on M17 medium. The morphology shows that *L. plantarum* Fibro 1 (Fig. 3D) is slightly larger compared to *L. rhamnosus* Fibro 2 (Fig. 3E) with lengths of 1.71 ± 0.35 μm and 1.39 ± 0.15 μm, and widths of 0.77 ± 0.21 μm and 0.52 ± 0.11 μm, respectively. Both strains like to grow on De Man, Rogosa and Sharpe growth media (MRS) and have inherent property to produce antimicrobial peptides (AMPs).

**Fig. 4:**
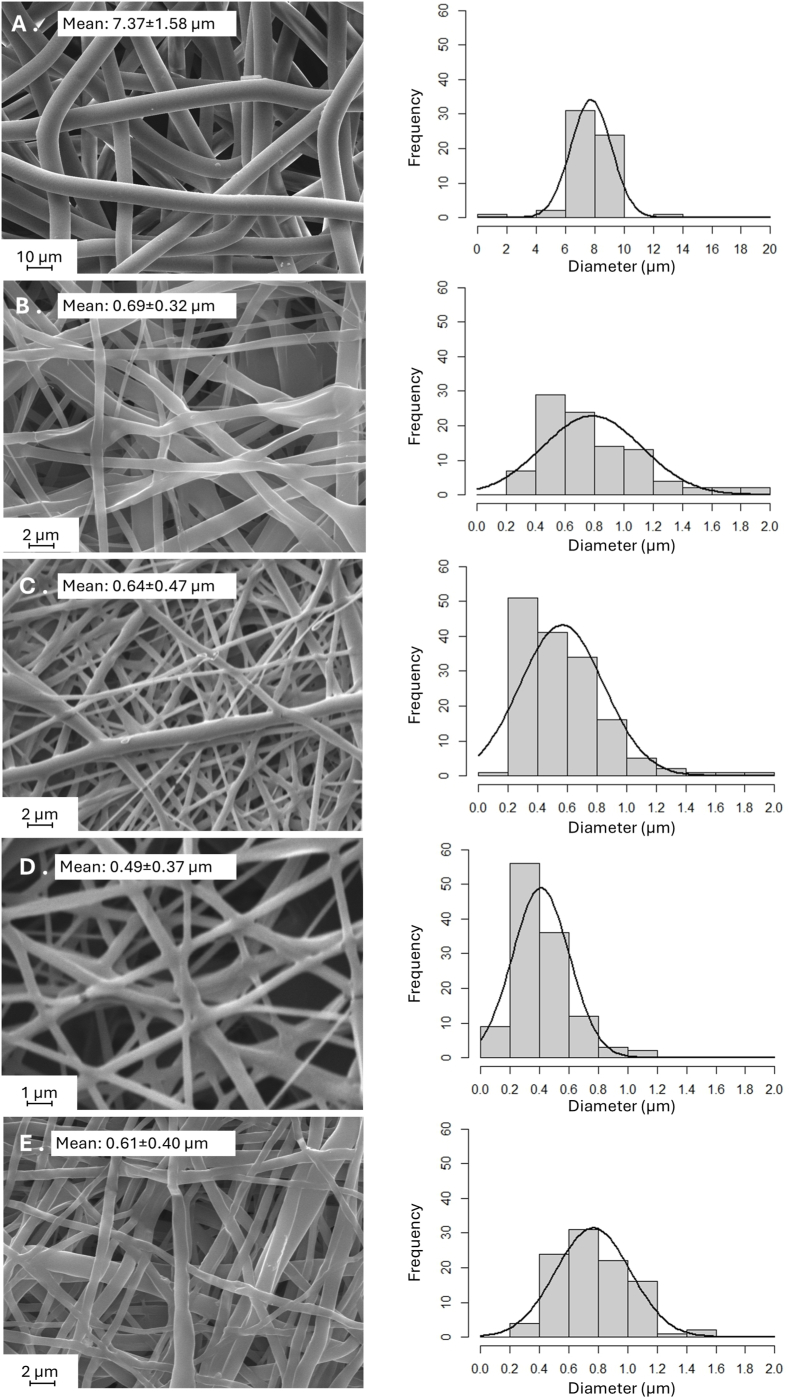
Scanning electron microscopy (SEM) micrographs showing the morphology of **A**. pristine PLC/PEO fibers (control), **B.** PLC/PEO fibers with agarose microcapsules (control) and PLC/PEO fibers LAB-loaded agarose microcapsules prepared via microchip electrospinning loaded with **C.***L. lactis* IL1403, **D.***L. plantarum* Fibro 1 and **E.***L. rhamnosus* Fibro 2.

LAB-loaded fiber matrices were produced similarly as the controls - LAB non-loaded agarose microcapsule-loaded fiber matrices. The microchip electrospinning process was not affected when LAB were incorporated into agarose aqueous solution prior electrospinning. LAB final concentration used for electrospinning was 10^8^ CFU/mL. SEM micrographs of pristine PLC/PEO electrospun fiber matrices and PLC/PEO fibers containing agarose microcapsules where different in size, but all these fibers can be called microfibers due to their large size (Fig. 4).

Pristine PLC/PEO fibers were the largest (Fig. 4A), the incorporation of agarose microcapsules into the fibers significantly reduced their diameter (Fig. 4B). Incorporation of different LAB (e.g. *L. lactis* IL1403, *L. plantarum* Fibro 1 and *L. rhamnosus* Fibro 2) into the microcapsules did not additionally affect the diameter of the fibers beging similar in size to the fibers consisting microcapsules without LAB (Fig. 4B *vs* **4C, D, F**).

The electrospinning process demonstrated high reproducibility across multiple batches. Pristine PLC/PEO fibers had more homogeneous diameters compared to those containing agarose microcapsules, despite whether loaded with LAB or not. The coefficient of variation (CV) in case of the pristine PLC/PEO was 21.4 %, compared to the CV of fibers with agarose microcapsules – 46.3 %. Among the samples with LAB, this value was 73.4 % for *L. lactis* IL1403, 65.6 % for *L. plantarum* Fibro 1, and 75.6 % for *L. rhamnosus* Fibro 2.

The image analysis of 2-dimensional (2-D) SEM micrographs were used to estimate the mean surface layer pore diameter. The largest pore size was observed in pristine PLC/PEO matrices (33.3 ± 37.0 μm^2^), although wide variation was present highlighting that also some smaller pores were detected (Table 1).

**Table 1:** Relevant properties of the electrospun fiber matrices. Data are presented as mean ± SD (N ≥ 3). Asterisks depict statistical significance of difference between without (PCL/PEO) and with agarose microcapsule-loaded samples: ∗ - p < 0.05; ∗∗ - p < 0.01; ∗∗∗ - p < 0.001; ∗∗∗∗- p < 0.0001; ns – p > 0.05. Key: LL – *L. lactis* IL1403, LP – *L. plantarum* Fibro 1, LR – *L. rhamnosus* Fibro 2.

	Pore size (μm)	Porosity (%)	Contact angle (°)
PLC/PEO	30.3 ± 37.0	20.8 ± 8.5	124 ± 7
PLC/PEO_agarose	0.90 ± 1.2 ∗∗∗∗	39.3 ± 7.3 ∗	88 ± 1∗∗∗∗
PLC/PEO_agarose_LL	0.7 ± 0.8 ∗∗∗∗	40.0 ± 4.3 ∗	108 ± 5 ∗
PLC/PEO_agarose_LP	0.9 ± 1.1 ∗∗∗∗	52.1 ± 8.8 ∗∗∗	86 ± 3 ∗∗∗∗
PLC/PEO_agarose_LR	0.8 ± 1.0 ∗∗∗∗	45.2 ± 5.0 ∗∗	84 ± 9 ∗∗∗∗

The incorporation of agarose significantly reduced the mean pore size to 0.9 ± 1.2 μm^2^, while further incorporation of lactic acid bacteria *(L. lactis* IL1403*, L. plantarum* Fibro 1*, L. rhamnosus* Fibro 2) did not markedly alter the pore size (Table 1). The porosity of the formulations containing agarose (with or without bacteria) is higher than that of the pristine PLC/PEO samples (Table 1). These findings are statistically significant, indicating that the fiber matrices with agarose are more breathable. This feature is essential in the wound healing process, as it facilitates oxygen exchange, which is critical for cell proliferation and tissue regeneration.

### 2.3 Wettability, mechanical properties and thickness of lactic acid bacteria-loaded fiber matrices

The contact angle of different electrospun matrices was measured at two time points: 0 s and at 30 s. The measurements showed that the pristine PLC/PEO fiber matrices were hydrophobic, with a contact angle of 124° (Table 1). Incorporation of low-melt agarose (with or without bacteria) significantly increased the hydrophilicity, reducing the contact angle (Table 1). No statistically significant differences were observed at the 30-s timepoint compared to the initial 0-s timepoint (Figure S4B, Supplementary information).

**Fig. 5:**
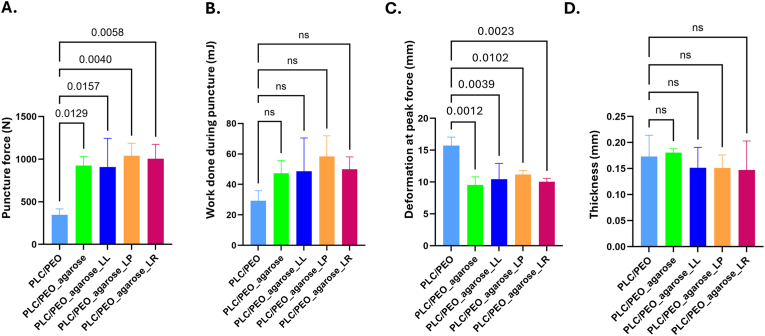
Mechanical properties and thickness of pristine PLC/PEO fiber matrices (PLC/PEO), agarose microcapsule-loaded PLC/PEO fiber matrices (PLC/PEO_agarose), PLC/PEO fibers containing microcapsules with LAB. **A.** Puncture force; **B.** Work done during puncture; **C.** Deformation at peak force; and **D.** Thickness. Key: LL – *L. lactis* IL1403, LP – *L. plantarum* Fibro 1, LR – *L. rhamnosus* Fibro 2. Data are presented as mean ± SD (N ≥ 3). Statistical significance: The p-values are shown above the significance brackets, ns – p > 0.05.

The prepared PLC/PEO fiber matrices loaded with agarose microcapsules (with or without LAB) exhibited higher hardness values compared to the fibers without microcapsules. All relevant mechanical properties measured (puncture force, deformation at peak force, and work done during puncture) showed that the presence of microcapsules within the fibers improved their mechanical properties (Fig. 5).

It is important to note that the thickness of all fiber matrices was similar, ranging from 0.14 mm to 0.18 mm, with a mean thickness of 0.17 ± 0.03 mm (Fig. 5D). The differences in mechanical behavior are not attributed to the varying thickness of the matrices but rather to their material composition and structure. This suggests that the microcapsule-loaded fibers are stronger and more resistant to puncture than the pristine PLC/PEO fiber matrices (Fig. 5A). The work done during puncture values, which reflect the energy required to deform or break the material, are negligible for the PLC/PEO fiber matrix, suggesting it is very soft and mechanically weak (Fig. 5B). In contrast, the fibers with agarose microcapsules have higher work done during puncture values, especially those of containing *L. plantarum* Fibro 1, indicating increased strength and toughness compared to pristine PLC/PEO fiber matrix. Interestingly, the PLC/PEO fiber matrix has the highest deformation values, which is due to lower resistance to localized deformation (i.e., lower puncture force). The other formulations, particularly those with *L. plantarum* Fibro 1 and *L. rhamnosus* Fibro 2, show lower deformation at peak force values, suggesting higher local stiffness and potentially offering more structural support compared to pristine PLC/10.13039/100017777PEO fiber matrix. This suggests that the PLC/PEO microfibers incorporating LAB-loaded agarose microparticles may serve as a suitable substrate or matrix for wound healing, as they can better withstand mechanical forces while maintaining relevant physical properties.

### 2.4 Number and viability of lactic acid bacteria in the fiber matrices

**Fig. 6:**
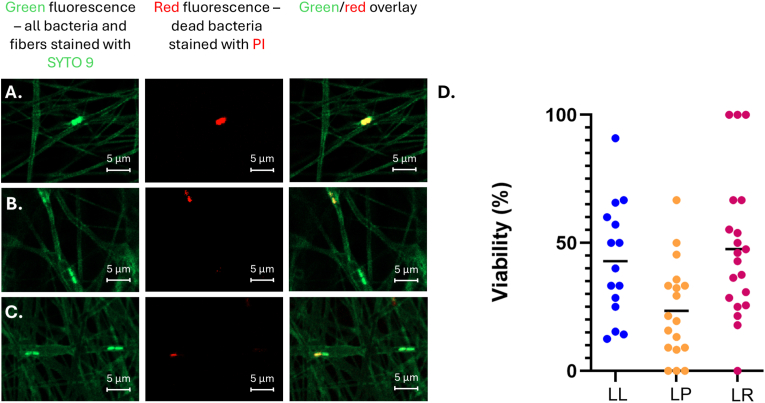
Representative zoom-in of confocal fluorescence microscopy (CFM) micrographs (with a size of original image 100 × 100 μm) of electrospun fibers containing agarose microcapsules with **A.***L. lactis* IL 1403, **B.***L. plantarum* Fibro 1, and **C.***L. rhamnosus* Fibro 2. The samples were stained after electrospinning using the live/dead stains SYTO 9 and propidium iodide (PI). A bacterial mixture (20 μL on a microscope slide), consisting of live (overnight liquid LAB culture) and dead LAB cells (heat-treated at 56 °C for 10 min) in a 50:50 ratio, was used as a control for imaging (data not shown). A 488 nm laser was used for transmitted light imaging as well as for excitation of the fluorophores. Green fluorescence images were acquired in the 493–558 nm range, representing live cells (SYTO 9), while red fluorescence images were captured in the 634–759 nm range, indicating dead cells (PI). **D.** The proportion (%) of viable LABs relative to the total number of LAB within the fibers. Key: scale bar: 5 μm, green – live bacteria and fibers (SYTO 9 stain), red – dead bacteria (PI); LL – *L. lactis* IL1403, LP – *L. plantarum* Fibro 1, LR – *L. rhamnosus* Fibro 2. (For interpretation of the references to color in this figure legend, the reader is referred to the Web version of this article.)

Usually, fibers are dissolved in a buffer and then analyzed by colony forming unit (CFU) counting if hydrophilic polymer matrices are used. However, in the case of hydrophobic polymer matrices different approaches need to be used. The prepared electrospun fibers with hydrophobic polymer contained living LAB and their viability and functionality were proven using live-dead assay and developed pH reduction evaluation method. One of the key aspects of the study was to confirm the viability of bacteria within electrospun PLC/PEO fibers and to determine how long the bacteria remain viable and metabolically active. For this purpose, samples were stained with SYTO 9 and PI and analyzed with CFM. The analysis of confocal Z-stack images revealed that LABs tested for electrospinning and encapsulation were present within the fibers and mostly viable LABs were observed, but also some non-viable bacteria were present (Fig. 6).

**Fig. 7:**
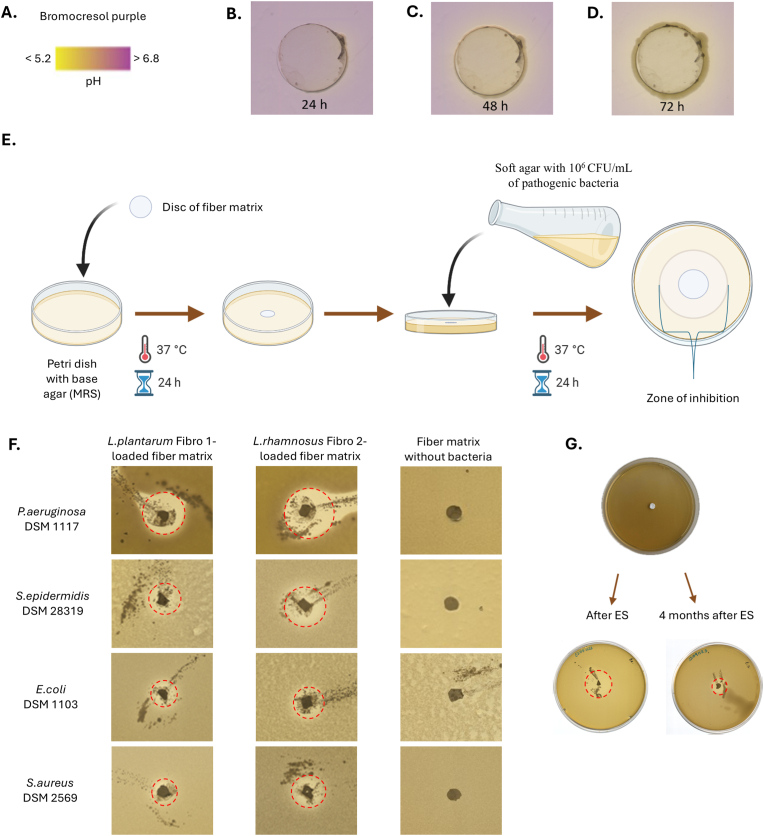
Viability and functionality (including antimicrobial activity) of probiotic bacteria within electrospun fiber matrices. **A.** Bromocresol purple indicator incorporated into M17 agarose plates was used to visualize the decrease of pH in the surrounding medium caused by probiotic (*L. rhamnosus* Fibro 2)-loaded fiber matrices electrospun directly onto glass discs. The color change of the media due to acidification is shown at different time points: **B.** 24 h, **C**. 48 h, **D**. 72 h. **E.** Schematics of agar overlay assay setup. **F.** Agar overlay assay using 6 mm diameter fiber matrix discs with and without different probiotic bacteria namely (*L. plantarum* Fibro 1 and *L. rhamnosus* Fibro 2). Initial concentration of pathogenic bacteria in the soft agar was 10^6^ CFU/mL. **G.** Schematics illustrating the *L. rhamnosus* Fibro 2-loaded fiber matrix disc, cut from the entire sample and placed on MRS base agar. The antimicrobial activity against *E. coli* DSM 1103 was assessed using the agar overlay assay immediately after electrospinning, as well as after 24 h and 4 months. Key**:** red dashed circles indicate zones of inhibition, representing the activity of probiotic bacteria against relevant pathogenic bacteria. (For interpretation of the references to color in this figure legend, the reader is referred to the Web version of this article.)

To determine the bacterial concentration in the fiber matrices, the number of LAB with green-fluorescent signal in the sample was quantified. The reproducibility of microchip electrospinning was tested over a minimum of three different days for each LAB, with the electrospinning process lasting 3 h for each fiber matrix. The total LAB concentration in the electrospun fiber matrix was approximately 10^8^ bacteria per 90 cm^2^ fiber matrix. It remained comparable to the initial bacterial dispersion concentration, indicating that no bacterial loss occurred during the electrospinning process. To determine the mean viability of LAB in the fiber matrix, the proportion of bacteria with a red fluorescent signal was subtracted from the number of bacteria with a green-fluorescent signal. The viability of *L. lactis* IL1403, *L. plantarum* Fibro 1 and *L. rhamnosus* Fibro 2 was 42.9 ± 22.6 %, 23.5 ± 18.8 %, and 52.4 ± 30.7 %, respectively (Fig. 6D). Specifically for L. *lactis,* additional viability analyses were performed in bacterial cell analysis pipeline 24 h after electrospinning and the viability of bacteria was confirmed (Fig. S3, Supporting Information). The results showed that bacteria remain viable in the fiber matrix in aqueous conditions up to 24 h. In addition to the live-dead staining, to prove the viability and functionality, the production of bacterial metabolite (e.g. lactic acid) was measured when LAB were grown on glucose as described in the literature [38]. These LAB produce lactic acid which lowers the pH of the surrounding environment. The higher the bacterial concentration and metabolic activity, the more visible is the yellow color of pH indicator surrounding the bacteria, indicating increased acid production (Fig. 7A). The method revealed that LAB, used in this experiment, produce lactic acid within microcapsules and microcapsule-loaded electrospun fibers already after 24 h, this metabolite is released into the surrounding environment as yellow circles were visible surrounding the electrospun fiber matrices (Fig. 7B). After 48 h (Fig. 7C) and 72 h (Fig. 7D) the size of changed colour zone increased together with increased intensity of yellow colour confirming the viability of bacteria and their functionality to produce lactic acid.

### 2.5 Antimicrobial activity of live probiotics-loaded fiber matrices

In previous experiments, various LABs, including *L.lactis* IL1403, *L.plantarum* Fibro 1, and *L.rhamnosus* Fibro 2, were incorporated into the fiber matrices. Among them, *L. lactis* IL1403 was initially selected as a model organism to demonstrate that different bacterial species can successfully be embedded into the fibers using this method. However, *L. lactis* IL1403 does not possess the ability to produce antimicrobial compounds, unlike *L. plantarum* Fibro 1 and *L. rhamnosus* Fibro 2, which (according to the manufacturer), are capable of synthesizing such bioactive substances. Therefore, following experiments focused exclusively on *L. plantarum* Fibro 1 and *L. rhamnosus* Fibro 2.

The antimicrobial activity of living *L. plantarum* Fibro 1 and *L. rhamnosus* Fibro 2 containing fiber matrices against a range of Gram positive and Gram-negative wound infecting bacteria (*E. coli* DSM 1103, *S. aureus* DSM 2569, *P. aeruginosa* DSM 1117, *S. epidermidis* DSM 28319) was determined by developed agar overlay assay (Fig. 7E). Clear inhibition zones were detected around the fiber matrices (Fig. 7F), and the diameter of the zone allows comparing the antibacterial efficacy of the probiotics-loaded electrospun fiber matrices against relevant wound pathogens as well as different probiotics with each other. Samples of fiber matrices were incubated on MRS agar for 24 h before coating with pathogen containing soft agar. After second 24 h incubation, it was seen that *L. plantarum* Fibro 1 and *L. rhamnosus* Fibro 2 both were able to produce substances that supressed all these pathogenic bacteria. The mean inhibition zone diameter measured for *L. plantarum* Fibro 1 was 12.6 ± 2.5 mm for *E. coli* DSM 1103. The respective number for *L. rhamnosus* Fibro 2 was 24.1 ± 5.5 mm. It is of interest and future studies enable to understand what are the effectors (lactic acid, AMP, etc) produced by the probiotic bacteria.

The antibacterial activity of *L. rhamnosus* Fibro 2 loaded fiber matrices were tested against *E. coli* DSM 1103 at different time points (immediately after electrospinning and after storage) in an agar overlay assay. It was confirmed that even 4 months after electrospinning (stored in the refrigerator at 4 °C) this probiotic bacterium was functional, and the fiber matrix retained its antimicrobial properties (Fig. 7G).

## 3 Discussion

Electrospinning is a relatively simple and convenient technology for producing micron-to-nanoscale fibers. It has been shown that in addition to various drugs, it is possible to incorporate into the fibers also living cells (e.g. bacterial cells) [26]. It offers a unique solution for encapsulating LABs due to the large specific surface area and high porosity of fibers [39]. Although a wide variety of polymers can be used for electrospinning, e.g. poly(acrylonitrile), poly(caprolactone), PLC, PEO, poly(vinyl pyrrolidone), poly(vinylidene fluoride), poly(vinyl alcohol) etc. [40] not all of these have been used for the encapsulation of living bacterial cells such as probiotics. Mainly water-soluble and biocompatible polymers together with aqueous solvents have been used [41]. For hydrophobic and water insoluble biocompatible polymers there are not many suitable solvents available that would not harm the LABs. Hydrophobic water insoluble polymers however enable the production of matrices which preserve their structure in aqueous environments and suit well for local wound healing applications. Due to the toxicity of organic solvents, strong acids or harsh electrospinning conditions (e.g. high voltage, drying), the LABs should be encapsulated to protect them from these effects, as the viability of the LABs, specifically of probiotics, is crucial for maintaining their health benefits.

Microfluidics can be used to encapsulate and protect the 10.13039/100026099LAB by forming a physical barrier around bacteria to support the cell structure and reduce contact with damaging conditions [27]. The use of microfluidics (microfluidic chips) alone for LAB's encapsulation is widely used [42], but it requires an additional final dosage form (e.g. dispersion, gel) development for the administration.

There are several electrospinning techniques that can protect bacteria from environmental stress and solvent toxicity and simultaneously produce suitable carrier matrices for delivery and application, such as coaxial electrospinning, emulsion and side-by-side electrospinning [25,43,44]. In these approaches, bacteria are typically embedded within dry polymer fibers, requiring sufficient moisture for reactivation. The produced structure of the fibers protects the probiotics (e.g. core-shell structure formed during coaxial and emulsion electrospinning). But these approaches still suffer from optimization, formulation and process setup challenges [21]. Therefore, in the present study, microchip electrospinning technology is proposed. The microchip electrospinning is an innovative method, that combines the accuracy of microfluidics with the flexibility of electrospinning to effectively encapsulate the LABs *in situ* (Fig. 1). In our method, the bacteria are encapsulated in semi-solid agarose microcapsules, which are then embedded within the fibers. The bacteria remain entrapped within the fibers, but thanks to the hydrophilic nature and dissolution of PEO, nanopores are formed and nutrients can reach the bacteria, and microbial metabolites can be secreted into the environment. The function of the microfluidic chip is not to achieve molecular mixing of two miscible phases. Instead, two immiscible phases are introduced into the T-junction: a continuous hydrophobic polymer solution and an aqueous agarose-bacterial dispersion as the dispersed phase. Therefore, no molecular mixing occurs within the chip; rather, the hydrodynamic conditions are adjusted to ensure reproducible formation of agarose microcapsules suspended in the polymer phase. The correct performance of the system was validated by microscopy, which confirmed homogeneous and stable microcapsule formation.

The incorporation of LAB into the agarose microcapsules *in situ* protects them from harsh environmental conditions including from drying out during the electrospinning process. We have shown that different bacterial species including relevant probiotic strains (namely *L. plantarum* Fibro 1, *L. rhamnosus* Fibro 2) having inherent capability to produce antimicrobial substances (e.g. AMPs, lactic acid) can be encapsulated in reasonable amounts into the fibers without major viability loss. As compared to traditional electrospinning where none of the bacteria survived the process, major advantages were seen with microchip electrospinning. The number of LABs which was successfully incorporated into 90 cm^2^-sized fiber matrix was approximately 10^8^ bacteria. It remained similar to the initial solution concentration (dispersion of LAB in an agarose solution before electrospinning), suggesting that no major bacterial loss took place during the electrospinning process. It has been shown previously that the viability of bacteria depends on the bacterial strain and species as different bacteria may show different resistance against mechanical and oxidative stresses and other potential stresses in their surrounding environment [25]. No major differences were observed between the three LAB species tested, but further studies are needed and will provide more insight into this topic.

After thorough preliminary experiments and formulation development studies, we selected PLC as a hydrophobic polymer and Me_2_CO_3_ as a suitable solvent and agarose for the microcapsule formation in our study. A hydrophobic PLC was chosen so that the dressing would not degrade fast in the wound area, thus ensuring the durability of the electrospun dressing during its use. However, our experiments showed that there was not enough release of chemical compounds out from and into these fibers (Fig. 3B). It was therefore necessary to add a hydrophilic polymer into the formulation, and PEO was chosen. The combination formulation of PLC and PEO was found to increase the nanoporosity of the fibers in the aqueous environment. The presence of agarose microcapsules influenced the mean diameter of PLC/PEO fibers, which were thinner than pristine PLC/PEO fiber diameters (Fig. 4). And it significantly affected the pore size between the fibers. The pristine PLC/PEO fiber matrices exhibited much larger pore sizes, with an mean of 30.3 ± 37.0 μm^2^, as determined by 2D upper-layer analysis of SEM images. After the addition of agarose (with or without bacteria), the mean pore size decreased, ranging from 0.7 ± 0.8 μm^2^ to 0.9 ± 1.2 μm^2^ (Table 1). The addition of LABs into the microcapsules did not change the morphology of the obtained fibers nor their fiber diameters or pore sizes between the fibers. Also the integration of agarose microcapsules significantly affected the mechanical properties of the fiber matrices (Fig. 5). PLC/PEO electrospun matrices incorporating agarose microcapsules (w/wo LAB) had higher puncture force values and lower deformation at peak force values, compared to the pristine PLC/PEO electrospun matrices. Thus, as a wound dressing, these electrospun matrices have the desired properties, such as the strength, lower deformation, and potential durability [45].

It is well-known that wettability of electrospun matrices is illustrating both the surface chemistry (hydrophobicity/hydrophilicity) as well as the roughness of the solid surface [46]. Wettability testing confirmed that our formulations containing agarose (with or without bacteria) possess advantageous characteristics for use in chronic and infectious wound environments. The pristine PLC/PEO formulation exhibited the highest contact angle (124°), indicating a hydrophobic surface (Table 1). Such hydrophobicity is beneficial for initial anti-adhesion, helping to prevent strong adherence of the dressing to moist wound surfaces [47]. However, the incorporation of low-melt agarose (with or without bacteria) increased surface hydrophilicity. This is beneficial in the treatment of chronic and infected wounds, because it indicates improved wettability and suggests enhanced potential for wound exudate absorption, without the excessive swelling and lateral expansion that can cause shear stress and damage to the periwound area [48], as observed in more highly absorptive dressings.

One of the most important results of this study was maintaining the viability and functionality of LABs during and after the electrospinning process. Integrating the probiotics into protective agarose microcapsules *in situ* before their incorporation into polymer fibers ensured that probiotics remained viable and functional (Fig. 6, Fig. 7). The viability and metabolic activity of the bacteria was demonstrated through live-dead staining and lactic acid production assays at different time points (24 h, 48 h, 72 h) (Fig. 7B–D). The study showed that nutrients can reach the encapsulated bacteria, and antimicrobial substances produced by the bacteria can diffuse out to cause effects on the surrounding environment. This two-way diffusion is essential for the sustained functionality of the probiotic bacteria and ensures continuous production of antimicrobial agents directly at the wound site, thus enhancing the wound healing process. This observation highlights the method's potential for creating effective wound dressings that can actively combat infections for extended time periods. More detailed investigations are planned for the future which allow comparisons between different probiotic strains in terms of their antimicrobial efficacy and potency. Also, it is shown that encapsulated probiotics retain their antimicrobial activity even after 4 months′ storage (Fig. 7G). It has been shown that probiotic *B. animalis* Bb12 encapsulated in polyvinyl alcohol electrospun fibers can retain viability up to 40 days stored at room temperature and up to 130 days stored under refrigerated conditions [49]. The study conducted by Hirsch et al. showed that *L. plantarum* encapsulated in polyvinyl alcohol-PEO fibers with a stabilizing excipient (skim milk) retained high viability, experiencing only a 0.2 log reduction was observed after one year of storage [50].

Our microchip electrospinning approach combines three key components: electrospun fibers, agarose-based microfluidic microcapsules, and probiotics. The resulting fiber matrices show potential to promote wound healing by providing high surface area, permeability, and porosity, which facilitate nutrient diffusion and compound release. The microcapsules and fibers protect probiotics from environmental stress and wound exudates. The method demonstrated good reproducibility, with consistent fiber diameters across batches, underscoring its potential for scalable clinical applications. Our results also show that the method can incorporate various bacterial species (*E. coli* Nissle 1917, *E. coli* MG1655, *E. coli* BW25113 - including genetically modified strains, *L. lactis* IL1403 [51], *L. plantarum* Fibro 1 and *L. rhamnosus* Fibro 2), into the fiber matrices (Fig. 1C). Agar overlay assay results confirmed that the embedded bacteria remain functional secreting bioactive compounds into the surrounding environment. This finding highlights the potential of using engineered model bacteria within the fibers to produce target compounds of interest. In the future it is possible to use these probiotic bacteria containing fiber matrices as functional wound dressings due to their sustained antimicrobial activity. Furthermore, as research in the field of engineered living biomaterials is evolving fast [3,52] our developed microchip electrospinning method can most probably also be used for the encapsulation of engineered living bacteria. Recent studies have demonstrated that probiotics-based dressings applied to the wound sites can enhance wound healing [53]. It is critical to evaluate the safety of such innovative probiotics delivery systems before they will be used on humans. Therefore, future studies will optimize the microchip electrospinning and evaluate the safety and *in vivo* efficacy of such probiotics-loaded fiber matrices.

## 4 Conclusions

We demonstrated that the microchip electrospinning method developed in this study offers a multifunctional platform for creating living probiotics-loaded fiber matrices. The method ensures high bacterial viability, improved mechanical properties and wettability, and provides sufficient substance diffusion and release. A comparison with relevant findings by other reports, we show that microchip electrospinning can overcome the limitations of traditional electrospinning by preserving a favorable microenvironment for probiotic bacteria. This approach is distinctive in its ability to protect probiotics from external factors being entrapped inside the fibers while simultaneously ensuring access to essential nutrients. As a result, it offers a novel route for engineering living materials with enhanced stability and function. Further *in vivo* experiments are needed to validate the therapeutic performance, and looking ahead, we envision extending this approach to a broader range of biomedical applications. Its distinctive properties—such as controlled release capability and biocompatibility—make it a highly promising platform for the development of smart wound dressings and other systems requiring sustained delivery of bioactive compounds.

## 5 Experimental section

*Materials, polymers, solvents*: For this research FDA approved polymers were used. As a continuous phase, copolymer of L-lactide and ε-Caprolactone in a 70/30 M ratio (PLC) was used as a suitable hydrophobic polymer (Purasorb PLC7015, Corbion, The Netherlands) and poly(ethylene oxide)(PEO)(SENTRY ™ POLYOX™ WSR 1105- LEO NF Grade, The Dow Chemical Company, MW 900 000 g/mol) was used as a hydrophilic polymer in some formulations together with PLC for testing their microchip electrospinning. As a dispersed phase, low melt agarose (AppliChem GmbH, Germany) aqueous solution was used. GRAS solvent was selected for microchip electrospinning - dimethyl carbonate (Me_2_CO_3_) obtained from ThermoFischer Scientific Inc., Germany. Glucose and lactose (Sigma-Aldrich, USA) were utilized as supplements for M17 bacterial growth medium. Glycerol (AppliChem GmbH, Germany) was used as a cryoprotectant for storing the bacterial stocks in the −80 °C freezer (pHcbi, Japan).

*Bacteria:* Different lactic acid bacteria (LAB) were used in the present study. Gram-positive bacteria *L. rhamnosus* Fibro 2 and *L. plantarum* Fibro 1 (Bioprox Healthcare, France) were used for the preparation of functional electrospun fiber matrices. According to the manufacturer's information, these lactobacilli inherently produce antimicrobial peptides (AMPs). In addition, *L. lactis* IL1403 (TF-TAK, Estonia), probiotic bacterium *E. coli Nissle 1917* (Mutaflor, Pharma-Zentrale GmbH, Germany) and various laboratory strains of *E. coli* (*E. coli* MG1655, *E. coli* BW25113) were used for the development of a microchip electrospinning method for the encapsulation of living probiotics. The preparation of genetically modified *E. coli* is described in the Supporting Information.

None of the probiotic bacteria were genetically modified and can be used in the final wound dressings. Gram-positive and gram-negative pathogenic bacteria isolated from wounds, namely *E. coli DSM 1103, S. aureus DSM 2569, P. aeruginosa DSM 1117, S. epidermidis DSM 28319* (DSMZ, Germany), were used to assess the antimicrobial activity of probiotic bacteria (e.g. LAB) within fiber matrices. All bacterial strains were maintained at −80 °C in an appropriate medium supplemented with 20 v/v% glycerol. They were revived in an appropriate medium prior to each experiment.

*Growth media and buffer media: L. lactis* IL1403 was incubated in BD Difco™ M17 broth (BD Bioscience, USA) with the addition of 0.5 w/v% of glucose, *L. rhamnosus* Fibro 2 and *L. plantarum* Fibro 1 were incubated in BD Difco™ MRS broth (BD Bioscience, USA). For the pathogenic bacteria BD Difco™ Luria-Bertani (LB) agar (BD Bioscience, USA) was used. In an agar overlay assay two different growth agars were utilized – MRS medium with 1.5 w/v% agar as a base and the top agar composed of BD Difco™ Mueller-Hinton II medium (Cation-Adjusted)(BD Bioscience, USA) with 0.5 w/v% of BD Difco™ Bacto agar (BD Biosciences, USA) and containing 10^6^ CFU/mL of pathogenic bacteria. For bacterial dilutions PBS buffer (pH 7.2)(Corning, USA) was used.

*Dyes:* Probiotic bacteria (free and encapsulated within fibers) were stained using green-fluorescent nucleic acid stain SYTO 9 and red-fluorescent nucleic acid stain propidium iodine, PI. All stains were purchased from Invitrogen, Thermo Fischer, USA. Also, bacteria were stained using red membrane stain FM 4–64 (Fisher scientific, Thermo Fischer, USA). As a pH indicator - bromocresol purple (Fisher scientific, Thermo Fischer, USA) was used in bacterial metabolic activity assay.

*Preparation of electrospinning solutions:* For the preparation of electrospinning solutions, pristine 12 w/w% PLC solution and 12 w/w% PLC/0.3 w/w% PEO solution in Me_2_CO_3_ were prepared. Other compositions were also tested during the development of probiotics-loaded fiber matrices (Supporting Information), but these were the final formulations used. Polymer solution was kept on a magnetic stirrer at room temperature, RT (21 ± 2 °C) for one-day prior use. PLC solution with PEO was heated on the heat-plate at 40 °C with constant stirring 150 RPM for 24 h prior electrospinning. Before electrospinning, the solution was cooled down to RT. Pristine PLC solution did not need additional heating. For the preparation of microcapsules, 0.625 w/w% agarose aqueous solution was prepared. 0.019 g of low melt agarose powder was weighed, and 3 g distilled water added. Agarose aqueous solutions were prepared in vials and left stirring on heated (45 ± 2 °C) magnetic stirrer overnight. The agarose solution was used as a dispersed phase and the microcapsule forming agent for both polymer solutions (single polymer vs two polymers).

*Preparation of bacterial suspension: L. lactis* IL1403 was streaked on the M17 agar plate with added 0.5 w/v% glucose and incubated at 30 °C overnight. *L. plantarum* Fibro 1 and *L. rhamnosus* Fibro 2 were streaked on the MRS agar and incubated at 37 °C overnight. After that one colony of this overnight grown culture was taken and inoculated into 10 mL of modified M17 broth (with the addition of glucose, final concentration of 0.5 w/v%) or MRS broth and incubated at 30 °C or at 37 °C overnight, respectively. The next day optical density (OD) of LAB was measured at OD_600_ with spectrophotometer (Shimadzu, UV-1800 UV–Vis, Japan). Then bacterial suspensions were dispersed in PBS buffer in Eppendorf tubes and centrifuged at 5000 RCF for 10 min. The medium was decanted, replaced with 0.625 w/w% low melt agarose solution and diluted to OD _600_ of 2. Pathogenic bacteria were streaked on LB agar plate and incubated at 37 °C overnight prior experiment. The next day few colonies were taken to make bacterial suspension with OD _600_ of 0.1.

*Viscosity of electrospinning solutions:* The viscosities of electrospinning solutions were measured with DVNext Cone&Plate rheometer (Brookfield, USA) using CP-52 spindle at 5 RPM (at ambient environmental conditions; temperature protocolled: 21.6 ± 0.6 °C). The volume of the sample was 0.5 mL.

*Microchip electrospinning:* Microchip electrospinning was used for the preparation of living probiotic-loaded fiber matrices. Microfluidic set-up consisted of a chip made of polydimethylsiloxane (PDMS) (self-made at the Institute of Fundamental Technological Research, Polish Academy of Sciences, Poland), its schematics shown in Fig. 1B and CAD file (.dwg) provided as a Supplementary file. The microchip was designed in a T-junction layout, featuring two inlet channels for the input fluids and a single outlet. All channels had a square cross-section with a standard width of 1 mm, except in the junction region, where the widths of all channels were reduced to 0.4 mm over a distance of 1, 2, or 4 mm from the center of the junction. This narrowed zone served as the microcapsule formation section, where two immiscible fluids met and spontaneously formed bacteria-loaded microcapsules suspended in a polymer solution (Fig. 1). The microcapsules formed in the T junction of the microchip were carried by the polymer solution flow (pointed out by an arrow in Fig. 1B) and exited from the outlet channel towards the needle attached to the exit tube.

Electrospinning was conducted using an electrospinning chamber (ESR200RD, NanoNC, South-Korea). Plastic syringes (3.5 mL, Nippro) were used and single use blunt needle B. Braun of 21G (inner diameter of 0.51 mm) was used. To produce homogenous microcapsules, two different flow rates were used for the two different polymer solutions. For the PLC solution and PLC/PEO polymer solution the flow rate was 0.9 mL/h and 0.2 mL/h flow rate was used for the probiotic bacterial dispersion. Electrospinning parameters used were: voltage of 13 kV and distance between the needle and collector 130 mm. The temperature during the electrospinning process was 29.1 ± 1.2 °C. To keep the relative humidity (RH) in the electrospinning chamber in specified ranges (7–12 %) dehumidifier (COTES A/S, Denmark) was used. These two parameters were maintained within these specified ranges to prevent clogging and keep the conditions optimal during the electrospinning process. The electrospinning electrode was attached to the metal needle and the fibers were collected on either a glass microscope slide (for microscopy images) or aluminum foil (non-sticky, Reynolds Consumer Products, USA) on a collector plate (30 × 30 cm). Microcapsule formation in microfluidic chip was monitored visually and by an optical camera. An optic microscope (Ceti, Medline Scientific, United Kingdom) was used to observe the fiber formation before the collection of larger fiber matrices. Ready electrospun samples (probiotic-loaded fiber matrices and pristine polymeric matrices) on foil were put into Ziplock bags before further analyses.

*Morphology characterization of electrospun fiber matrices:* Fiber morphology and fiber size analyses were performed using scanning electron microscopy (SEM) (Zeiss EVO® 15 MA, Germany). The samples were sputter coated using approximately 3 nm thick platinum layer.

*Wettability of the matrices:* To understand the hydrophilic/hydrophobic nature of the fiber matrices and their wettability behavior, the contact angle was measured by the sessile drop method (OCA 15 EC, DataPhysics Instruments GmbH, Filderstadt, Germany). A drop (5 μL) of 1 × PBS buffer solution (pH 7.4) was applied onto the fiber matrices (size 2 × 2 cm). The contact angle measurements were taken at time points 0 and 30 s after the liquid drop touched the surface of the fiber matrix. This test was carried out at RT (22 °C ± 0.1). The contact angle was analyzed using SCA20 software (DataPhysics Instruments GmbH, Filderstadt, Germany). Each sample was measured at least in triplicate.

*Mean porosity and pore size between the fibers:* To quantify the mean porosity of the fiber matrices, three independent batches were fabricated for each formulation. From each fiber matrix, three SEM micrographs were acquired, and image analysis of the upper layers was performed in ImageJ software to determine the total pore area. It was then used to estimate the porosity by dividing the total pore area by the total micrograph area, expressing porosity as the percentage of void space within the upper layer of fiber matrix according to modified method of Ting Wang et al. [54]. In addition, the pore size between the fibers was measured and mean pore size calculated from the upper layer of the 2D SEM micrographs.

*Mechanical analyses:* The mechanical behaviour of the probiotic-loaded and unloaded fiber matrices was studied by Brookfield CT3 Texture Analyzer (Middleboro, MA, USA) equipped with a 10 kg load cell. Puncture test was performed using TexturePro CT software (AMTEK Brookfield, Middleboro, MA, USA). 2 × 2 cm pieces were used for mechanical analysis which were secured between the film support fixture (TA-FSF) and punctures were made with a cylinder probe (TA-42, diameter 3 mm). The target distance of 40 mm was used with all the samples with trigger load of 5 g and test speed of 2.5 mm/s. All measurements were performed at ambient conditions (temperature of 21.1 ± 0.7 °C and RH of 20 ± 2 %). Each sample group comprised of at least 5 specimens. The applied force (N) and distance of the probe (mm) were recorded as the probe deformed the sample and puncture force (N), deformation at peak force (mm) and work done during puncture (mJ) were calculated in the TexturePro CT software (AMTEK Brookfield, Middleboro, MA, USA). The thickness of the samples was measured using Precision-Micrometer 533.501 (Scalamesszeuge, Dettingen, Germany) with a resolution of 0.01 mm.

*Storage stability of electrospun fiber matrices:* To investigate the impact of storage conditions on the morphology of the electrospun probiotics-loaded fiber matrices and the viability of the incorporated LAB, samples from one batch were stored at RT (21 ± 2 °C) in a desiccator with 0 % RH over silica gel to prevent humidity-induced changes in the matrices, and in a refrigerator at 4 °C in ziplock bags. During specified time-points the morphology was investigated using SEM and viability using agar-overlay assays.

*Release and diffusion of small molecules in and out from the electrospun fibers:* The transport of substances in and out from the electrospun fibers containing microcapsules was proven using confocal fluorescence microscopy (10.13039/100021544CFM)(Zeiss 10.13039/501100009542LSM 710; Germany) and nucleic acid stain SYTO 9 and the data analyzed using developed bacterial cell analysis pipeline (Table S2, Supporting Information). The diffusion of green-fluorescent nucleic acid stain (SYTO-9) into the microcapsule loaded fibers was investigated by color change (from red to green). Briefly, collected fiber matrices containing *L. lactis* IL1403 (pre-stained with FM 4–64 prior electrospinning) loaded agarose microcapsules on microscopy cover glass were put onto the 1 % agarose pads consisting of glucose, covered with another cover glass and kept at 30 °C for specified time periods (24 h). These samples were initially measured directly under the CFM (confirmed the red colored bacteria within fibers) and then the cover glass was removed and SYTO 9 stain added (1 μL stain per 1 mL bacterial dispersion). After 30 min these stained samples were again measured under the microscopy. The staining of bacteria within the fibers with SYTO 9 and its efficacy enabled to prove whether the stain can diffuse into the fibers or not. CFM micrographs with a size of 67.48 × 67.48 μm were used for further analysis.

*Viability of LAB:* The presence, distribution, overall concentration and viability of 10.13039/100026099LAB within the electrospun fibers was investigated using staining with fluorescent nucleic acid stains PI and SYTO 9 and 10.13039/100021544CFM (Table S2, Supporting Information). The electrospun fiber matrices containing LAB-loaded agarose microcapsules were collected on a microscopy cover glass and then were put onto the 1 % agarose pads consisting of M17-glucose medium, covered with another cover glass (Fig. S1, Supporting Information) and analyzed immediately or incubated at 30 °C or at 37 °C, for 24 h. For staining 20 μL of 10 μM SYTO 9 and 20 μL of 40 μM PI were added to the agarose pad (opposite side from the fibers). Diffusion of dyes was allowed to take place at least 20 min before imaging. A bacterial mixture consisting of live (overnight liquid LAB bacterial culture) and dead LAB (heat treated at 56 °C for 10 min) in 50:50 ratio was used as a control for imaging. These samples were photographed with the CFM (LSM710, Carl Zeiss, Germany). Z-stack images with a size of 100 × 100 μm micrographs were collected with 488 nm laser excitation and emission in green and red channels. Zen software was used for the analysis (Carl Zeiss Microscopy GmbH, Germany). During the analysis of the Z stack images (on average, 10 μm in depth), the live bacteria (green) and total bacteria (red and green) were counted. The average viability of LAB in fiber matrix was calculated using the following formula (Equation 1):

[Equation 1] Viability %= (number of live bacteria/ total number of bacteria) × 100 %

*Bacterial metabolite lactic acid production on agar plate (pH reduction assay):* The viability and functionality of LAB within the fibers was measured by the production of bacterial metabolite lactic acid. The latter was measured when LAB were grown on the M17 1 % agarose plates with glucose (final concentration of 0.5 w/v %) and with pH indicator bromocresol purple (Fisher scientific, Thermo Fischer, USA) (4.6 mg of indicator in 200 mL medium). pH indicator changes its colour in different pH conditions (from purple to yellow in acidic environment). Probiotic-loaded fiber matrices electrospun on small glass slides (12 mm in diameter) were put onto these agarose plates and kept at 30 °C (for *L. lactis* IL1403) and at 37 °C (for *L.plantarum* Fibro 1 and *L. rhamnosus* Fibro 2) for specified time periods (24 h, 48 h, 72 h) before analysis. At each time point images were collected by scanner (Epson, Japan).

*Antimicrobial activity of live probiotic bacteria containing fiber matrices:* The antimicrobial activity of fiber matrices containing live LAB against a range of Gram-positive and Gram-negative pathogenic bacteria isolated from wounds *(E. coli DSM 1103, S. aureus DSM 2569, P. aeruginosa DSM 1117, S. epidermidis DSM 28319*) was determined using an agar overlay assay modified from the method of Lokman Hossain et al. (2022) [55]. A 6 mm fiber matrix disc was put on a Petri dish containing 15 mL base agar (MRS agar) and incubated at 37 °C for 24 h. A suspension of pathogenic bacteria was prepared with OD_600_ 0.1 and diluted 1:100 in soft agar (Mueller-Hinton medium with 0.5 w/v% agar). After that 10 mL of soft agar with pathogenic bacteria was poured over the base agar and let to solidify. After solidification Petri dishes were kept at 37 °C for 24 h. Analysis was performed in triplicate.

*Statistical analysis:* To evaluate the homogeneity of the fibers, a coefficient of variation (CV) - a measure of the relative variability of data, expressed as a percentage – was calculated using Equation (2).

[Equation 2] CV=(σ/μ) × 100 %

Where.

• σ is the standard deviation of the dataset.
• μ is the mean of the dataset.

A one-way ANOVA was conducted to determine whether differences in the composition of electrospun formulations had a significant effect on the quantitative parameters (p < 0.05). To calculate the arithmetic mean values and standard deviations, all tests were performed at least three times. P-values were adjusted using the Bonferroni correction. The statistical analyses were performed using GraphPad Prism version 10.0.3 (GraphPad Software, USA, www.graphpad.com), and differences with a p value ≤ 0.05 were considered significant. Drawn images were created with BioRender (www.biorender.com). Graphs were constructed with GraphPad Prism version 10.0.3 (GraphPad Software, USA, www.graphpad.com). Microscopy images were visualized with ZEN 3.8 (Carl Zeiss Microscopy GmbH, Germany). The statistical measures and visualizations for bacterial cell pipeline analyses were obtained in Python, using the Seaborn, Matplotlib for visuals and SciPy f for statistical analyses. The Welch *t*-test was used to compare the bacteria in PLC mats with the bacteria in PLC/PEO matrices. The statistical significance threshold was set to 0.05. The diameters of the inhibition zones were measured using the ImageJ program (National Institutes of Health, USA).

## CRediT authorship contribution statement

**Oksana Gerulis:** Writing – original draft, Visualization, Validation, Methodology, Investigation, Formal analysis, Data curation, Conceptualization. **Georg-Marten Lanno:** Writing – review & editing, Visualization, Validation, Methodology, Investigation, Formal analysis, Data curation, Conceptualization. **Marta Putrinš:** Writing – review & editing, Visualization, Validation, Supervision, Methodology, Investigation, Formal analysis, Data curation, Conceptualization. **Marilin Moor:** Writing – review & editing, Visualization, Validation, Software, Methodology, Investigation, Formal analysis, Data curation. **Beata Niemczyk-Soczynska:** Writing – review & editing, Methodology, Investigation, Formal analysis. **Tomasz Kowalczyk:** Writing – review & editing, Supervision, Resources, Methodology, Investigation, Funding acquisition, Conceptualization. **Slawomir Blonski:** Writing – review & editing, Methodology, Investigation. **Tanel Tenson:** Writing – review & editing, Supervision, Resources, Methodology, Conceptualization. **Piotr M. Korczyk:** Writing – review & editing, Resources, Methodology, Investigation. **Karin Kogermann:** Writing – review & editing, Visualization, Validation, Supervision, Resources, Project administration, Methodology, Investigation, Funding acquisition, Formal analysis, Data curation, Conceptualization.

## Declaration of competing interest

The authors declare the following financial interests/personal relationships which may be considered as potential competing interests:

Karin Kogermann reports a relationship with EsaDres that includes: board membership and equity or stocks. Marta Putrins reports a relationship with EsaDres that includes: equity or stocks. Tanel Tenson reports a relationship with EsaDres that includes: equity or stocks. Karin Kogermann has patent #PCT/EP2022/076874 pending, Georg-Marten Lanno has patent #PCT/EP2022/076874 pending, Marta Putrinš has patent #PCT/EP2022/076874 pending, Tanel Tenson has patent #PCT/EP2022/076874 pending, Piotr M. Korczyk has patent #PCT/EP2022/076874 pending, Tomasz Kowalczyk has patent #PCT/EP2022/076874 pending, Slawomir Blonski has patent #PCT/EP2022/076874 pending. If there are other authors, they declare that they have no known competing financial interests or personal relationships that could have appeared to influence the work reported in this paper.

## Data Availability

We have uploaded most of the data as Supplementary material and microscopy images are provided using DataDOI data repositorium link (https://doi.org/10.23673/re-541). Additonal data will be made available on request.
