# Supplementary material for: Living probiotics-loaded wound matrices prepared by microchip electrospinning
Source: Mater Today Bio. 2025 Oct 10;35:102403. doi: 10.1016/j.mtbio.2025.102403 (PMC12550174; doi:10.1016/j.mtbio.2025.102403)
Supplement: Multimedia component 1 [file mmc1.pdf]

Supporting Information for

Living Probiotic-Loaded Wound Matrices Prepared by Microchip Electrospinning

*Oksana Gerulis, Georg-Marten Lanno, Marta Putrinš, Marilin Moor, Beata Niemczyk-Soczynska, Tomasz Kowalczyk, Slawomir Blonski, Tanel Tenson, Piotr Korczyk, Karin Kogermann**

Oksana Gerulis, Georg-Marten Lanno, Marta Putrinš, Marilin Moor, Karin Kogermann*
Institute of Pharmacy, University of Tartu, Nooruse 1, 50411 Tartu, Estonia
E-mail: karin.kogermann@ut.ee

Beata Niemczyk-Soczynska, Tomasz Kowalczyk, Slawomir Blonski, Piotr Korczyk
Institute of Fundamental Technological Research Polish Academy of Sciences, Pawinskiego 5B, 02-106 Warsawa, Poland

Tanel Tenson
Institute of Technology, University of Tartu, Nooruse 1, 50411 Tartu, Estonia

*Corresponding author: K. Kogermann, Institute of Pharmacy, University of Tartu, karin.kogermann@ut.ee ; ORCID: 0000-0002-6813-4828

Materials and Methods

Preparation of genetically modified E. coli

Genetically modified *E. coli* strains were modified with different plasmids to make the bacteria to produce fluorescent proteins (GFP- green protein, mCherry- red protein, BFP- blue protein) which enabled to visualize the bacteria within the polymer fiber matrices.

Microfluidic chip preparation:

For preliminary experiments, different microfluidic chips were prepared using different designs (3 channel/cross-junction, 2 channel/T-junction) and materials (polycarbonate, PDMS). The three channel system was made from polycarbonate with inlet channels of square cross-section of the size of 0.4 mm x 0.4 mm narrowing to 0.1 mm x 0.1 mm cross-section at the junction, acting as a microcapsule forming section. This system was used for Na-alginate microcapsule preparation. Two channel chip made from PDMS was initially used for the preparation of agarose microcapsules in Me_2CO_3. PDMS had a channel cross-sectional dimension of 1 mm and microcapsule forming section of 0.4 mm. The lengths of constricted channels forming a T-junction for microcapsule formation finally selected were 1 x 1 x 1cm, although also other designs were tested. For microchip electrospinning, three-channel (two inlet and one outlet) PDMS chips were prepared.

Sample preparation for confocal fluorescence microscopy (CFM) analysis:

Samples for CFM were prepared as shown on **Figure S1**.

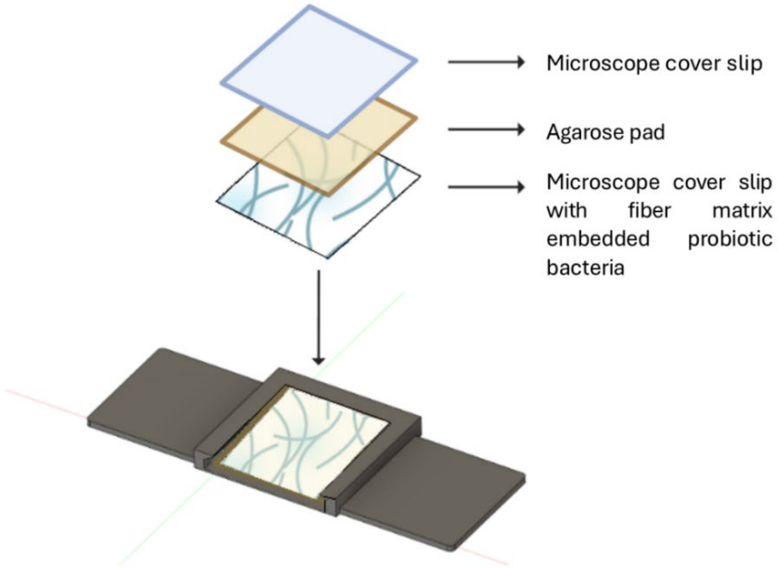

Figure S1. Schematic illustration of sample preparation for confocal fluorescence microscopy (CFM) to investigate the viability and presence of probiotic bacteria encapsulated within electrospun fibers

Fibers loaded with microcapsules containing bacteria were electrospun onto microscopy cover glass. Agarose pad preparation: 800 μL of a soluble 1% agarose solution (M17 media + lactose + 0.5 w/v% glucose) was pipetted onto another cover glass. After solidification, the microscopy glass containing fibers was placed on the prepared agarose pad. For staining, 20 μL of 10 μM SYTO 9 and 20 μL of 40 μM PI were added to the agarose pad (on the opposite side of the fibers), and diffusion of the dyes was allowed to take place for at least 20 min before imaging. Cover glasses with the agarose pad were then placed into a microscopy glass-sized plastic holder.

Preliminary experiments for final formulation development:

Microcapsule preparation:

Microcapsules were made using the microfluidics chips with various designs for different formulations (s.f. *Microfluidic chip preparation*). Initially, Na-alginate was tested for making microcapsules via ionic cross-linking previously described (US Patent US20200069726A1). 1 w/w% and 2 w/w% of Na-alginate aqueous solutions were prepared and stirred until homogeneous solution was obtained. The aqueous solution of $CaCl_2$ (2.5 w/w%) was prepared and served as an ionic crosslinking agent in alginate gelation process (Na-alginate solutions was dripped into $CaCl_2$ solution). Various oil phases (vaseline, sunflower, almond oil) were used as continuous phase mixed with surfactant in different amounts (sorbitan monooleate SPAN80, 5-10 w/v%). The best results were obtained using a formulation of 1 w/w% Na-alginate, almond oil/SPAN 80 (5 w/v%) and 2.5 w/w% $CaCl_2$ (3-channel polycarbonate microfluidic chip). Secondly, hexadecane solution instead of oil phase with 2 w/w% SPAN80 and 1 w/w% Na-alginate was tested. Thirdly, agarose aqueous solution (1-2 w/w% concentrations) was tested for microcapsule formation and hexadecan as a solvent by collecting the formed microcapsules into the ice-cold vial. The best formulation was hexadecan together with 2 w/w% SPAN 80 and 1 w/w% agarose aqueous solution. Optimised flow rates for different channels in microfluidic chamber providing stable microcapsule formation were 1 mL/h for oil/hexadecane and 0.1 mL/h for agarose solution. Fourthly, agarose solution (1-2 w/w% concentrations) was tested together with Me_2CO_3 as a solvent. Flow rates for microcapsule formation were 0.5 mL/h for Me_2CO_3 and 0.1 - 1 mL/h for agarose solutions. And final formulations tested for microcapsule formation in microfluidic chamber were polymer

PLC solutions (5 w/w% and 10 w/w%) in Me_2CO_3 and agarose solutions with different concentrations. Flow rates varied from 0.2 to 2 mL/h for both phases.

Microcapsule purification: microcapsules were formed using prepared microfluidic chips and the selected formulations, but these consisted of a lot of oil phase and/or solvents which can harm living bacteria. The filtration and purification of these microcapsules were conducted and vacuum filtering with water rinsing enabled to remove most of the continuous phase. The results confirmed the need to conduct *in situ* microchip electrospinning directly from a microfluidic chip and avoid any intermediate steps.

Selection of solvents for *microchip electrospinning:*

Various other solvents were tested for their suitability to be used together with the PDMS microfluidic chip (**Table S1**).

Table S1. Solvents tested for the formulation development using microchip electrospinning

Solvent(s)	Solvent volume [mL]	Solvent mass [g]	PDMS mass before experiment [g]	PDMS mass after 24 h solvent exposure [g]	Swelling [%]	PDMS swelling factor	Water miscibility	PLC solubility	Potential for use
Dimethyl carbonate (Me_2CO_3)	3.000	3.200	0.152	0.175	15.1	A	-	+	+
Chloroform ($CHCl_3$)	3.000	4.120	0.149	0.451	202.7	SE	-	+	+
Hexafluoroisopropanol (1) (HFIP)	3.000	4.630	0.149	0.206	38.3	NO	+	+	-
Dimethylformamide (DMF)	3.000	2.956	0.173	0.177	2.5	NE	+	-	-

Acetone (Me_2CO)	3.000	2.180	0.148	0.185	24.7	A	+	-	-
Dimethylsulfoxide (DMSO)	3.000	3.356	0.201	0.206	2.4	NE	+	-	-
Dichloromethane (CH_2Cl_2)	3.000	3.720	0.165	0.347	109.7	S	-	+	+
Ethyl acetate ($CH_3CO_2CH_3$)	3.000	2.660	0.198	0.321	61.5	A	-	-	-
Acetonitrile (CH_3CN)	3.000	2.340	0.172	0.177	2.6	NE	?	?	?
Dichloroethane (CH_2ClCH_2Cl)	3.000	3.718	0.208	0.336	61.8	S	-	?	-
Diethyl carbonate (Et_2CO_3)	3.000	2.955	0.198	0.287	44.9	A	-	-	-
N-butyl lactate (NBL)	3.000	2.966	0.148	0.165	11.5	NE	-	-	-

Propyl lactate (PL)	1.500	1.550	0.172	0.184	6.977	NE	?	-	-

(1) - time of contact with a solvent – 48h, Key: A - acceptable, NE - neglible, NO - noticeable, SE - severe, S – strong; +- yes; - - no; ?- not possible to identify

Although also some other solvents showed acceptability with the PDMS solvent and no major changes to the chip integrity, Me_2CO_3 was selected to be the final solvent for microchip electrospinning. Mainly due to its suitability with the PDMS chip, fast evaporation rates and reported lower toxicity to bacterial cells.[1]

Bacterial cell analysis pipeline:

Bacterial cell analysis pipeline was developed for the analysing the fluorescence information from CFM micrographs. This was achieved by converting the CZI files to a stack of red and green channel images. The images were then used for counting bacteria automatically. This was accomplished by implementing several image processing methods. Briefly, the images were uniformly resized, thresholded, morphologically operated with disk-shaped kernels. This allowed to count the bacteria for all the stacks.

This created pipeline was used for the analysis of *L. lactis* loaded fiber matrices (with two different compositions: PLC fibers and PLC/PEO fiber matrices) and the pipeline was used for understanding the diffusion of SYTO 9 stain into the different fiber formulations and determining the viability of bacteria within the fibers (**Table S2**).

Table S2. Dataset used for the bacterial cell analysis pipeline.

Dataset	Assay	Bacteria	Additional staining after ES
24 CZI files (14 with PEO)	Effect of PEO on the fiber permeability	*L. lactis* pre-stained with FM 4-64 (red dye)	SYTO 9 (green dye)

25 CZI files (7 with 24h)	Bacteria viability in fiber	*L. lactis*	SYTO 9 (green dye) + PI (red dye) at 0th hour or 24th hour

Keys: CZI file- Carl Zeiss Image files, the native file format of the ZEN software by Carl Zeiss Microscopy GmbH., ES- electrospinning, FM 4-64- red stain that binds cell membranes, PI- propidium iodide is a nucleic acid intercalating dye (red), used for visualization of dead cells, SYTO 9- green-fluorescent nucleic acid stain, visualization of all cells.

24 CZI files (image size 67.48 x 67.48 µm) of *L. lactis*-loaded fiber matrices were used for the analysis. *L. lactis* was pre-stained with FM4-64 (red) and stained with SYTO 9 (green) and the aim was to understand whether the diffusion of DNA stain SYTO 9 is taking place and whether the addition of PEO into the formulation increases the diffusion of stain into the PLC fibers. In order to support or reject the null hypothesis, the percentage of SYTO 9 stained bacteria per sample was found for matrices with PLC and PLC/PEO.

In addition, by using CFM micrographs of *L. lactis*-loaded fiber matrices (25 CZI files of *L. lactis*-loaded fiber matrices stained with SYTO 9 (green) and propidium iodine PI (red))(image size 67.48 x 67.48 µm), the viability of bacteria within fibers was analysed. The question if bacteria were viable within the fiber was answered through estimating the amount of green (alive) bacteria to the amount of red (dead) bacteria throughout all the planes in one CZI file. The statistical measures and visualizations were obtained in Python, using the Seaborn, Matplotlib for visuals and NumPy for calculations.

For this assay the *L. lactis* bacteria were stained with SYTO 9 and PI, where PI (red dye) was a signal of dead bacteria. The number of green bacteria (SYTO 9 stained) to dead bacteria (PI-stained) were determined. The viability of the strains within the fiber matrix was also determined 24 h after electrospinning, when the matrix was incubated in bacterial growth enabling conditions. **Figure S2** shows that after incubation, the bacteria remain viable, and the amount of live bacteria relative to the total number slightly increased.

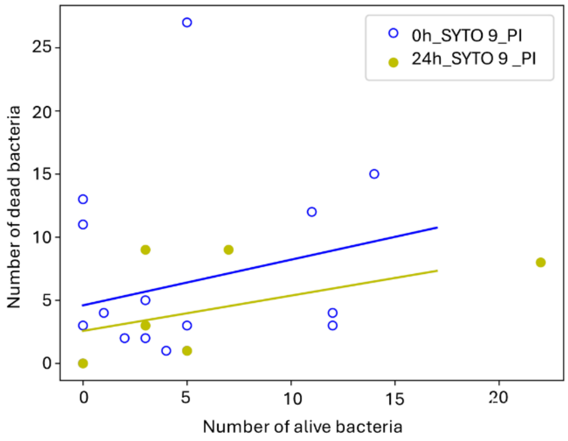

Figure S2. The amount of viable *L. lactis* IL1403 relative to the total number of bacteria within the fibers, which were SYTO 9-PI-stained immediately after electrospinning and 24 h of incubation in growth enabling conditions. Key. LL – *L. lactis* IL1403; PI- propidium iodide is a nucleic acid intercalating dye (red), used for visualization of dead cells, SYTO 9- green-fluorescent nucleic acid stain, visualization of all cells.

Dye diffusion assay:

Preliminary testing of the diffusion of substances out from the electrospun fibers was measured using blue food dye (Sky blue, UK). The electrospun fiber matrices were prepared using the agarose microcapsules-loaded 15 w/w% PLC formulations, but instead of bacteria, food dye was added into the agarose solution. Firstly, the fiber matrices were put onto LB plates and the diffusion of dye was monitored (**Figure S3**). Secondly, the diffusion of dye into the buffer solution was evaluated by immersion of samples into phosphate buffer solution (pH 7.4) and the absorbance was measured using microtiter plate reader at two different wavelengths (410 and 630 nm). The release of substances from microcapsules and electrospun fibers consisting of microcapsules enables to prove the transport of substances in and out of the microcapsules and microcapsule-loaded fibers. It is the prerequisite for providing conditions for bacteria to survive and also to collect the substances produced by bacteria within such systems.

The release of blue food dye from the microcapsules and microcapsule-loaded fibers was tested. Microcapsules consisting of 15 w/w% PLC in Me_2CO_3 + 0.625 w/w% agarose (+ 10 µL/mL food dye) were prepared, collected on LB agar plate and the release of dye was investigated. The droplet was released from the syringe tip when approximately 10 microcapsules were formed and formed a larger droplet. 6 separate samples were collected on LB plates. The test duration was 72 h, and even after this period, the droplets remained unchanged due to the formation of PLC film between agar and microcapsule droplet not allowing food dye to diffuse (**Figure S3**).

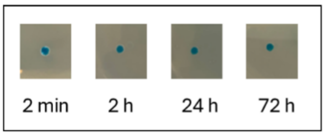

Figure S3. 15 w/w% PLC (Me2CO3) and 0.625 w/w% agarose microcapsules containing blue food dye (Blue Sky, UK) showing the dye release on the LB plate.

LB plates were stored at 37 °C and after 2 h two more samples had released the dye; others remained the same. After 24 h of incubation, the food dye had diffused altogether from 5 samples. After 72 h of incubation all samples had released some amount of blue food dye. This method confirms that the formed microcapsules are able to release the substances from their interior. The release is dependent on the contact between microcapsule and formation of PLC film, the latter inhibits the release of substances. The release of blue food dye into the buffer solution was also tested with electrospun microcapsule-loaded fibers (15 w/w% PLC in Me_2CO_3 + 0.625 w/w% agarose (+ 10 µL/mL blue food dye). For this experiment, the electrospun fiber samples were put into the buffer solution and the release of blue food dye from electrospun fiber matrices was measured by measuring the absorbance at 410 and 630 nm. It was seen that blue dye was released very slowly from the matrix into the buffer solution within the tested timeframe. Due to the desired wound infection treatment and healing application where the antibacterial efficacy can be obtained only when the release kinetics is carefully controlled (not too slow and not too fast), we made more investigations via using fluorescent dye diffusion into the fibers as described in the *Results* section. All these diffusion/release experiments proved that increased nanoporosity of the electrospun fibers consisting of microcapsules was achieved using slightly modified formulations and electrospinning conditions. There are various approaches that can be used to modify the release from fibers such as increased nanoporosity with the use of porogens (e.g. PEG/PEO-hydrophilic polymer) or solvent mixtures together with high humidity, [2] the use of alternative polymers. Formulation composition was modified by adding hydrophilic polymer PEO as a porogen into the PLC fibers to increase the nanoporosity and consequently the diffusion of molecules through the fibers. Different formulations were tested by varying the PLC as well as PEO concentrations and ratios. The addition of PEO directly to PLC15 w/w% solution resulted in too viscous solutions, and electrospinning was not possible (tested 0.5 w/w%, and 0.1 w/w% PEO addition). Concentrations varying between PLC 10 w/w% + PEO 0.1 w/w% in Me_2CO_3

and PLC 13 w/w% + 0.3 w/w% PEO in Me_2CO_3 worked for microchip electrospinning in our setup. It was seen that the addition of PEO within PLC layer enables to increase the release of blue dye from the microcapsule loaded electrospun fibers.

Contact angle determination:

To investigate the wettability properties of the fiber matrices, contact angle was determined. The contact angle of different electrospun matrices was measured at two time points: 0 s and at 30 s. Results showed that the addition of agarose microcapsules changed the wettability properties of electrospun matrices, and more hydrophilic fibers were obtained compared to PLC/PEO fiber matrices without agarose microcapsules (**Figure S4A**).

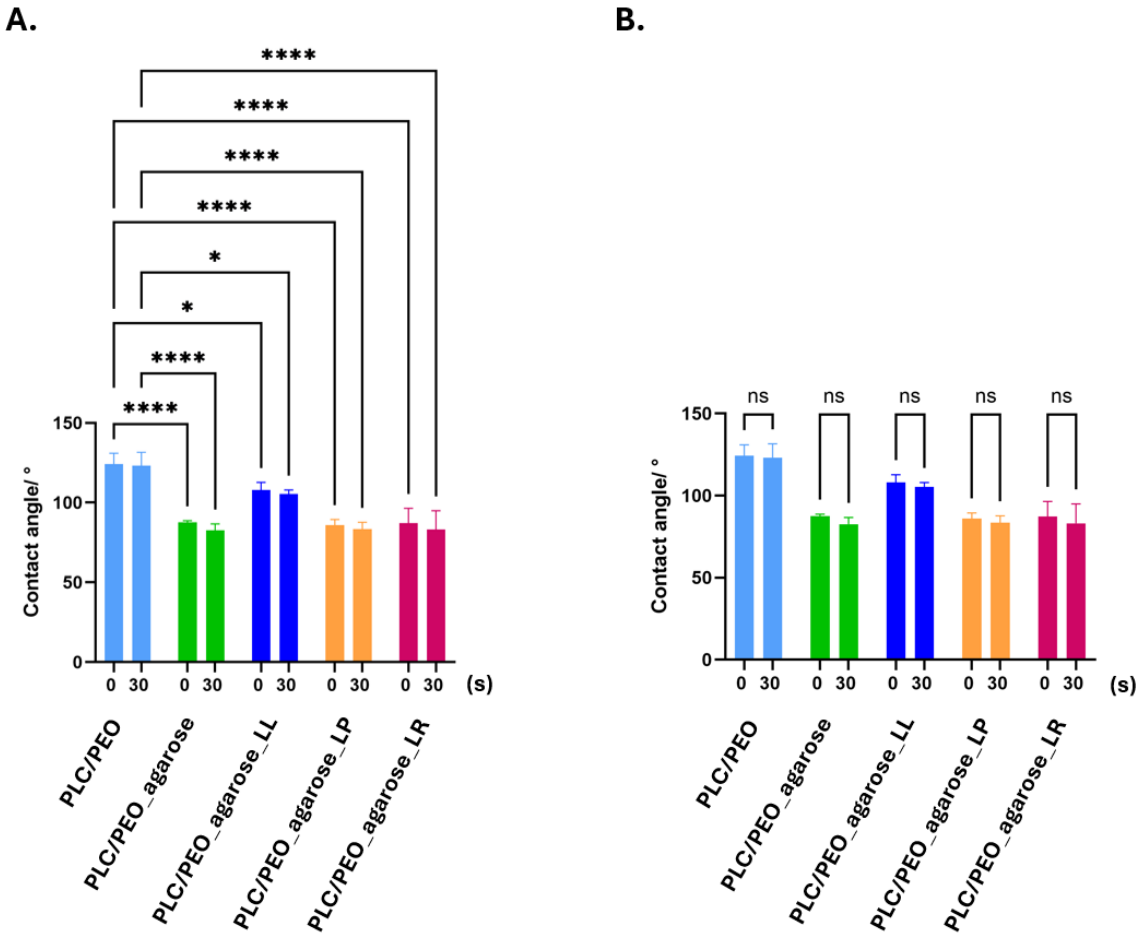

Figure S4. Contact angle measurements of fiber matrices at two time points, 0 seconds and 30 seconds, showing: **A.** Statistical differences between different sample types, and **B.** No statistically significant differences within the same sample type between 0 seconds and 30 seconds. Key: LL – *L. lactis* IL1403, LP – *L. plantarum* Fibro 1, LR – *L. rhamnosus* Fibro 2.

Data are presented as mean ± SD ($N \geq 3$). Statistical significance * - $p < 0.05$; ** - $p < 0.01$; *** - $p < 0.001$; ****- $p < 0.0001$; ns – $p > 0.05$.

No statistically significant differences in contact angle values were detected when measurements were performed at 0 s and 30 s timepoints (**Figure S4B**).

References

[1] E. Berthier, E.W.K. Young, D. Beebe, Engineers are from PDMS-land, biologists are from Polystyrenia, *Lab Chip* 12 (2012) 1224–1237. https://doi.org/10.1039/C2LC20982A

[2] C. Ramos, G.M. Lanno, I. Laidmäe, A. Meos, R. Härmas, K. Kogermann, High humidity electrospinning of porous fibers for tuning the release of drug delivery systems, *Int. J. Polym. Mater. Polym. Biomater.* 70 (2021) 880–892. https://doi.org/10.1080/00914037.2020.1765361.
